# Cognitive Flexibility Training Improves Extinction Retention Memory and Enhances Cortical Dopamine With and Without Traumatic Stress Exposure

**DOI:** 10.3389/fnbeh.2019.00024

**Published:** 2019-03-01

**Authors:** Lauren E. Chaby, Klevis Karavidha, Michael J. Lisieski, Shane A. Perrine, Israel Liberzon

**Affiliations:** ^1^Department of Psychiatry and Behavioral Neurosciences, Wayne State University School of Medicine, Detroit, MI, United States; ^2^Research Service, John D. Dingell VA Medical Center, Detroit, MI, United States; ^3^Department of Psychiatry, VA Medical Center, Ann Arbor, MI, United States; ^4^Department of Psychiatry, University of Michigan, Ann Arbor, MI, United States

**Keywords:** cognitive flexibility, dopamine, norepinephrine, single prolonged stress, trauma, PTSD

## Abstract

Stress exposure can cause lasting changes in cognition, but certain individual traits, such as cognitive flexibility, have been shown to reduce the degree, duration, or severity of cognitive changes following stress. Both stress and cognitive flexibility training affect decision making by modulating monoamine signaling. Here, we test the role cognitive flexibility training, and high vs. low cognitive flexibility at the individual level, in attenuating stress-induced changes in memory and monoamine levels using the single prolonged stress (SPS) rodent model of traumatic stress in male Sprague-Dawley rats. Exposure to SPS can heighten fear responses to conditioned cues (i.e., freezing) after a fear association has been extinguished, referred to as a deficit in extinction retention. This deficit is thought to reflect an impairment in context processing that is characteristic of posttraumatic stress disorder (PTSD). During a cognitive flexibility training we assessed individual variability in cognitive skills and conditioned rats to discriminately use cues in their environment. We found that cognitive flexibility training, alone or followed by SPS exposure, accelerated extinction learning and decreased fear responses over time during extinction retention testing, compared with rats not given cognitive flexibility training. These findings suggest that cognitive flexibility training may improve context processing in individuals with and without traumatic stress exposure. Individual performance during the reversal phase of the cognitive flexibility training predicted subsequent context processing; individuals with high reversal performance exhibited a faster decrease in freezing responses during extinction retention testing. Thus, high reversal performance predicted enhanced retention of extinction learning over time and suggests that cognitive flexibility training may be a strategy to promote context processing. In a brain region vital for maintaining cognitive flexibility and fear suppression, the prelimbic cortex (PLC), cognitive flexibility training also lastingly enhanced dopamine (DA) and norepinephrine (NE) levels, in animals with and without traumatic stress exposure. In contrast, cognitive flexibility training prior to traumatic stress exposure decreased levels of DA and its metabolites in the striatum, a region mediating reflexive decision making. Overall, our results suggest that cognitive flexibility training can provide lasting benefits by enhancing extinction retention, a hallmark cognitive effect of trauma, and prelimbic DA, which can maintain flexibility across changing contexts.

## Highlights

- Extinction retention after trauma was enhanced by prior cognitive flexibility training.
- Cognitive flexibility training may rescue cognitive deficits in PTSD.
- Individuals with high reversal learning performance had greater extinction retention.
- Cognitive flexibility training increased dopamine in the prelimbic cortex.
- Cognitive flexibility training buffered the effects of stress on striatal dopamine.

## Introduction

Maintaining cognitive flexibility, i.e., the capacity to shift behavioral strategies in a changing environment, is critical to an individual’s ability to update environmental representations (reviewed in Kehagia et al., 2010). Low cognitive flexibility can be precipitated by stress exposure, and variability in cognitive flexibility is high across and within species (Laughlin et al., 2011; Miyake and Friedman, 2012). In humans, retrospective clinical studies have shown that individuals with low cognitive flexibility exhibit increased psychopathology severity or progression, and deficits in cognitive flexibility have been characterized in affective, anxiety, and neurodegenerative disorders (Chamberlain et al., 2006; Dickstein et al., 2007; Tchanturia et al., 2012, 2013; Brockmeyer et al., 2014). For example, individuals with low cognitive flexibility have higher levels of posttraumatic stress disorder (PTSD) symptoms and less posttraumatic growth and optimism (Keith et al., 2015). Conversely, individuals with heightened cognitive flexibility may have enhanced resilience to change and self-efficacy (Kim and Omizo, 2005; Genet and Siemer, 2011; Mealer et al., 2012; Romero-Martínez et al., 2013). Cognitive flexibility can be heightened through interventions in childhood or adulthood (Masley et al., 2009; Moore and Malinowski, 2009; Genet and Siemer, 2011; Lewis-Morrarty et al., 2012). Investigating whether enhancement in cognitive flexibility, through cognitive training prior to trauma exposure, can reduce PTSD symptoms following trauma, could advance discussions of interventions for resilience and recovery from traumatic experiences.

Cognitive flexibility training paradigms often incorporate multiple aspects of cognitive flexibility, which all require “letting go” of an old association and acquisition of a new association, and extensive efforts have been made to identify psychological and neuropharmacological mechanisms underpinning aspects of cognitive flexibility (Kehagia et al., 2010). Two distinct aspects of cognitive flexibility are reversal learning, when reinforcement is shifted from a familiar, previously rewarded to cue to a familiar cue that was not previously rewarded, and attention shifting, where novel cues are presented and an individual forms an association with a new unconditioned cue (Birrell and Brown, 2000; Klanker et al., 2013). Animals undergoing reversal learning exhibit greater and more extended dopamine (DA) release in the medial PFC, compared with associative learning, but no difference in norepinephrine (NE) output (van der Meulen et al., 2007). Yet, tonic elevation of NE in the mPFC can enhance reversal learning and attention-shifting (Lapiz and Morilak, 2006). Thus, behaviorally distinct mechanistic aspects of cognitive flexibility share common regional specificity, including corticostriatal circuitry, and reliance on monoamine signaling (Logue and Gould, 2014), perhaps because of shared need for extinction of a familiar association and acquisition of a novel association.

Research in rodents and humans suggests that cognitive flexibility is mediated by reciprocal interactions between the striatum and PFC (reviewed in Klanker et al., 2013). For example, increased DA activity in the striatum can decrease DA in the PFC and limit PFC afferent input into the striatum (Roberts et al., 1994; Strafella et al., 2001; Goto and Grace, 2005). Striatal DA activity regulates inhibitory input, which acts on corticostriatal circuitry to affect cognitive flexibility (Logue and Gould, 2014). Similarly, PFC α_2_-adrenergic receptor binding density has a linear relationship with perseverative errors during a set shifting task (Arnsten et al., 1999). However, excess extracellular NE binds to α_1_-adrenergic receptors, which can broadly impair executive functions through dysregulation in cortical circuits (Arnsten et al., 1999; Carr et al., 2007; Arnsten, 2015; Luo et al., 2014, 2015). Severe, trauma-like stress can dampen mPFC activation and prefrontal glutamate levels (Knox et al., 2010; Perrine et al., 2016), but may enhance striatal activity and monoamine release (Abercrombie et al., 1989; Jastreboff et al., 2011; Nikolova et al., 2012). Together these reciprocal changes might suggest that, stress exposure can prompt a transition from reflective, flexible responding, mediated by the PFC to compulsive, reflexive responses mediated by the striatum (Keller et al., 1983; Sinha et al., 2005; reviewed in Arnsten et al., 2015).

Individual characteristics, including stress history, cause variation in aspects of cognitive flexibility, and the monoamine regulation of cognitive performance (Dias-Ferreira et al., 2009; Laughlin et al., 2011; Naegeli et al., 2013). Animal models that use longitudinal stress manipulations have been essential for the understanding of intersections between mechanisms that maintain cognitive flexibility and effects of trauma. For example, exposure to a rodent model of PTSD called single prolonged stress (SPS) can increase perseverative errors during reversal learning and never-reinforced errors during attention-shifting as well as dampen striatal DA signaling (Eagle et al., 2013; Enman et al., 2015; George et al., 2015). Exposure to SPS can also cause a cognitive deficit in rodents similar to that detected in PTSD patients, in which recall of fear extinction is impaired, called an extinction retention deficit (Milad et al., 2008, 2009; Knox et al., 2012, 2016; Perrine et al., 2016). Extinction retention can be impaired by reduced mPFC activation, loss of dopaminergic neurons in the mPFC, or disrupted DA signaling (Espejo, 2003; Mueller et al., 2010; reviewed in Greco and Liberzon, 2016). Indeed, rats exposed to SPS show reduced DA receptor density, DA levels, and DA metabolites in the striatum (Enman et al., 2015; Perrine et al., 2016). Conversely, completing reversal learning tasks can increase DA efflux in the mPFC (van der Meulen et al., 2007), while attention shifting tasks can increase DA efflux in the mPFC and dorsal striatum (Stefani and Moghaddam, 2006).

Given that cognitive flexibility can increase resilience and lessen PTSD symptom severity, it may mitigate effects of trauma by interacting with mechanisms directly dysregulated in PTSD (Goto and Grace, 2005; Moore and Malinowski, 2009; Lewis-Morrarty et al., 2012). Here, we investigate whether exposure to cognitive flexibility training can attenuate the effects of trauma on extinction retention of conditioned fear learning. To do this, we used SPS as a rodent model of traumatic stress that has been shown to diminish both extinction retention and cognitive flexibility performance (Knox et al., 2012, 2016; George et al., 2015). To address interacting mechanistic effects of cognitive flexibility training and stress exposure, we investigate corticostriatal monoamine and metabolite levels and their relationship with individual cognitive flexibility performance. We hypothesized that cognitive flexibility training would enhance resilience to the effects of trauma on behavior and catecholamine signaling.

## Materials and Methods

### Subjects and Housing

**Figure 1:**
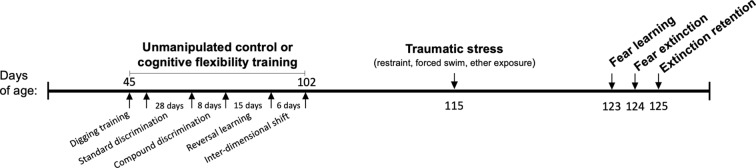
Timeline of procedures; DA, dopamine; NE, norepinephrine.

Male Sprague-Dawley rats (*n* = 36) were obtained at 40 days of age from Charles River Laboratories (Kingston, NY, USA). Upon arrival, rats were pair housed and randomly assigned to one of four possible treatments, with/without cognitive flexibility training and with/without exposure to SPS. Animals were housed in standard microisolator, plastic cages (20 × 26 × 45 cm) with wood chip bedding replaced weekly, and were given 5 days to acclimate following transport before experimental procedures began. A timeline of all procedures is depicted in Figure 1. Standard rat chow (LabDiet^®^ 5001, 23% protein) and tap water were available *ad libitum*, except prior to behavioral testing procedures that were rewarded, in these cases rats were food deprived for 2 h beforehand. Rats were kept at 20–22°C and 50% relative humidity on a 12:12 light/dark cycle. To control for circadian rhythms, tests were started a minimum of 3 h after the beginning of the dark cycle and completed within 4 h of the start of the test. Control rats and cognitive flexibility training rats received a weekly handling and weighing session until SPS procedures, to ensure that rats habituated to handling and maintained healthy weight. Following SPS, all rats were singly housed and were handled only for fear learning procedures described below (Liberzon et al., 1999 and Knox et al., 2012). Testing order was pseudo-randomized; and treatment groups were evenly distributed during the first and last hours of the testing. Equipment was sprayed with 70% ethanol in water solution and wiped clean between all trials and subjects. Experiments were approved by the VA Ann Arbor Healthcare System Institutional Animal Care and Use Committee (#1312-004).

### Cognitive Flexibility Training

A group of 18 rats were exposed to cognitive flexibility procedures based on Birrell and Brown (2000); one rat was removed because it would not consistently sample available food bowls. To ensure that potential effects of the cognitive flexibility training would persist, there was a 2-week delay following training, before a subset of eight rats were exposed to SPS as described below. The remaining nine rats exposed to cognitive flexibility training served as an unstressed control group. An additional 18 rats, not exposed to cognitive flexibility training, were maintained in standard housing conditions as controls until 115 days of age, when nine rats were exposed to SPS, and the remaining nine were used as unstressed controls.

#### Cognitive Flexibility Apparatus

The plastic testing apparatus (45 × 63 × 36 cm) contained a removable divider and two ceramic bowls, each in a corner opposite the start area (bowl diameter 7 cm, depth 4 cm). Both bowls contained 40 mL of digging substrate, in one bowl the substrate concealed a Honey Nut Cheerio^®^. Stock digging substrates contained 5.5 L of bedding mixed with 70 mL of an added scent cue (Table 1) and 10 Cheerios ground into powder, to balance reward scent cues. A removable divider separated rats from the bowls at the beginning of each trial, and was replaced after an error to prevent access to the rewarded bowl. The bowls used in the in-cage digging training and the cognitive flexibility training were identical.

**Table 1:** Cues used for cognitive flexibility tasks.

Odor pairs	Digging substrate pairs
Coriander, Cumin	Paper bedding, Clay
Cinnamon, Turmeric	Coconut husks, Corncob
Mustard, Fenugreek	Cellulose fiber, Confetti crinkles bedding

#### Digging Training

Laboratory rats can be trained to dig for food reward in the substrates listed in Table 2. To train for digging behavior, rats were individually presented with a bowl containing one-quarter of a Cheerio in clean cages identical to their home cage for 10 min on five occasion following 2 h of food deprivation. Digging bowls were identical to the cognitive flexibility training bowls. In the first session, the reward was presented in the empty bowl. The second session was identical to the first except that 10 ml of aspen bedding was added to the bowl, such that the Cheerio reward was still visible but partially obscured by bedding. An additional 10 ml of aspen bedding was added in each of the three subsequent Cheerio presentations, completely covering the reward, such that on the final exposure the bowl contained the same amount of digging substrate as in the cognitive flexibility trials (40 mL total).

**Table 2:** Descriptions of cognitive flexibility tasks.

Task	Description	Example cue reward pairing
Standard discrimination	Associative learning between a food reward and a cue, with only one cue type	Rewarded bowl—cinnamon Unrewarded bowl—turmeric (aspen bedding digging substrate)
Compound discrimination	Associative learning between a food reward and a cue, with two cue types: one relevant and one irrelevant cue type	Rewarded bowl—cinnamon (+ paper bedding or clay digging substrate) Unrewarded bowl—turmeric (+ paper bedding or clay digging substrate)
Reversal learning	The relevant cue is switched, but is within the same cue type	Rewarded bowl—turmeric (+ paper bedding or clay digging substrate) Unrewarded bowl—cinnamon (+ paper bedding or clay digging substrate)
Inter-dimensional shift	All available cues change; the relevant cue changes, within the same cue type	Rewarded bowl—mustard (+ cellulose or confetti digging substrate) Unrewarded bowl—fenugreek (+ cellulose or confetti digging substrate)

#### Cognitive Flexibility Training

A trial was initiated 20 s after the rat was placed into the “start area” of the arena by raising the removable divider to give the rat access to the reward bowl. In the first four trials, rats could dig in both bowls, but only one was rewarded. In subsequent trials, after a rat selected a bowl by inserting a nose or paw into the bowl, a plastic insert was immediately added to separate the animal from accessing the other chamber containing the unselected bowl. A trial was recorded as correct if the rat inserted its nose or paw into the rewarded bowl first. After a bowl was selected, the removable divider was inserted to prevent access to the unselected bowl, and the rat was allowed 20 s with the selected bowl to either consume the reward or reinforce the error. Rats were then transferred from the maze into a holding chamber for a 20 s intertrial-interval and the bowls were reset. If a rat did not select a bowl in 5 min, the animal was transferred from the maze into a holding chamber and the bowls were reset. The side of the arena containing the rewarded bowl was randomized, and bowls could be distinguished by multiple cues types. Each session contained four trials, sessions were conducted once each day during the four tasks comprising the cognitive flexibility training: a standard discrimination task (28 sessions), a compound discrimination task (eight sessions), a reversal learning task (15 sessions), and an inter-dimensional shift task (six sessions).

In the standard discrimination task, one cue type distinguished the rewarded and unrewarded bowl (associative learning; Table 1). For the compound discrimination task, a second cue type was introduced, but the cues from the standard discrimination task remained constant for eight additional training sessions (associative learning with distracting cues). For the reversal task, the context cues were not changed but the food-paired cue was switched, within the relevant cue type, such that the previously incorrect cue became the relevant cue. For the inter-dimensional shift task, the cues in the relevant and irrelevant cue types were all changed (a total change in all cues), and a novel cue from the previously-relevant cue type signaled the reward location. To maximize contrast between digging substrate textures and reduce the degrees of freedom, cues were always used in pairs; for example, if clay contained the rewarded stimulus, the unrewarded stimulus was always paper bedding, and vice versa (Birrell and Brown, 2000). The order of the tasks was always the same, but the cues were equally represented within groups and counterbalanced between groups so that an equal number of rats from the SPS and unstressed group were exposed to each cue pairing.

### Single Prolonged Stress

The SPS rodent model of traumatic stress exposure was used here because it has been used for two decades to model PTSD-specific traits (Liberzon et al., 1997; Khan and Liberzon, 2004; reviewed in Lisieski et al., 2018). Similar to PTSD patients, exposure to SPS can increase GR receptor levels, startle responsivity, anxiety-like behavior, pro-inflammatory cytokine levels, sleep disturbances, anhedonia, and cause extinction retention deficits (Khan and Liberzon, 2004; Yamamoto et al., 2009; Nedelcovych et al., 2015; Vanderheyden et al., 2015; Lin et al., 2016; reviewed in Deslauriers et al., 2018). In the SPS model, rats are exposed to three stressors in succession (lasting approximately 3 h in total), followed by social isolation for 7 days, procedures described in Khan and Liberzon (2004) and Knox et al. (2012). Rats were first restrained for 2 h, then underwent 20 min of forced swim in cold water (23–24°C), in a 68 × 56 × 45 cm opaque plastic container. After swimming, rats were dried and given 15 min to recuperate. Next, rats were exposed to ether vapors in a desiccator until loss of consciousness, as determined by lack of a paw withdrawal or toe pinch reflex response. Animals were then individually-housed in clean cages and left undisturbed for 7 days, the delay required for a PTSD-like phenotype to develop (Liberzon et al., 1999; Knox et al., 2012). Prior research has demonstrated that SPS-induced neuroendocrine effects, including HPA negative feedback and glucocorticoid receptor mRNA expression, are only evident after SPS following a 7-day quiescent period (Liberzon et al., 1997, 1999), reflecting the 30-day post-trauma delay required before PTSD can be diagnosed in humans (reviewed in Cahill and Pontoski, 2005). Thus, it is often included in the SPS model to isolate rodents for a 7-day quiescent period to prevent social buffering, and to integrate lasting effects of the trauma exposure (Knox et al., 2012; Chen et al., 2018; George et al., 2018). As brief periods of isolation in adulthood can also have neuroendocrine effects, control rats were also isolated to account for potential effects of housing (Raz and Berger, 2010).

### Fear Learning

After the 7-day quiescent period following SPS, rats were trained to associate a tone with a shock across five shock-tone pairings in a fear conditioning chamber (day 1: fear conditioning). Then, in a novel context, rats were repeatedly presented with the same tone until their fear responses to the tone were extinguished (day 2: fear extinction). Finally, rats were returned to the second context and re-exposed to the tone to determine if they retained the information that the tone was not paired with the shock in the second context (day 3: extinction retention). The fear conditioning context was distinguished from the second context using visual, olfactory, and tactile cues (additional details in Supplementary Methods). Freezing responses were quantified as a proxy of fear (Bouton and Bolles, 1980; Knox et al., 2012); freezing was defined as immobility, lasting longer than 1 s, but allowing for small pendulum-like head movements with all four feet and the body immobile (and without vibrissae flicking), as this is also suggested to be a fear behavior in rats (Kolpakov et al., 1977) and other small mammals (Halpin, 1983; Ayon et al., 2017). To minimize disturbance, the experimenter was not in the room during testing; trials were video recorded and freezing behavior was measured by analysists blind to treatment. Freezing behavior was measured in temporal blocks defined by each stimulus presentation, then percent time freezing was calculated as (time freezing in stimulus block/total time in stimulus block) × 100.

#### Fear Learning: Shock Reactivity

To determine if the cognitive flexibility treatment affected pain sensitivity, behavioral response to the five shocks administered during fear conditioning was rated by two independent analysts blind to treatment conditions, using video recordings to allow for later analysis. Shock responses were rated on a 5-point scale, modified from Menard et al. (2004): (1) flinch involving only the head or forepaw; (2) whole body flinch, with or without ambulation; (3) whole body flinch and/or jump (all four feet in the air), followed by ambulation, or a jump without ambulation; (4) whole body flinch, followed by running; and (5) whole body jump (all four feet in the air), followed by running.

### Neurochemical Analysis With High-Pressure Liquid Chromatography (HPLC)

On the day following extinction retention procedures, brains were harvested following rapid decapitation and flash frozen for later processing. To obtain brain region tissue punches, brains were thawed at −20°C for 10 min. Brains were then sliced using a chilled stainless steel rat brain matrix, resulting 2 mm sections were mounted on dry ice. Using a 1.5 mm biopsy punch, bilateral tissue punches were obtained from the prelimbic cortex (PLC), infralimbic cortex, and dorsal anterior striatum, in accordance with the Paxinos and Watson Rat Brain Atlas. Tissue punches were transferred to microcentrifuge tubes and frozen at −80°C for subsequent analysis.

Tissue punches were suspended in 50 μL of 0.2 N HClO_4_, then sonically disrupted and centrifuged at 4°C and 12,300 rotations per minute for 10 min. Then, a 25 μL aliquot of the resulting supernatant was obtained from each sample, and monoamine analysis was performed on a Dionex Ultimate 3,000 high-pressure liquid chromatography (HPLC) system (Thermoscientific, Waltham, MA, USA), equipped with an autosampler maintained at 4°C, which autoinjected 10 μL of sample into a 100 μL sample loop on a C18-RP (2 μL diameter) column maintained at 25°C. Thermoscientific TEST Mobile Phase flowed in the column at a rate of 0.6 mL/min, and contained acetonitrile, phosphate buffer, and an ion-pairing reagent; coulometric electrochemical detection was achieved with a dual electrode cell set at −175 mV (reference) and 300 mV (working). Chromatograms were analyzed using Dionex Chromeleon software (version 7); a detection threshold was set at three times the average height of four solvent peaks (neurochemicals below this threshold were omitted from further analysis). Absolute values of monoamines (DA, NE) and monoamine metabolites (DOPAC, 3,4-dihydroxyphenylacetic acid; HVA, homovanillic acid; 3MT, 3-methoxytyramine) were determined by comparison with five dilutions of external standard (Sigma-Aldrich, St. Louis, MO, USA) run in parallel and in duplicate, once at the beginning and once at the end of each run. Monoamine and metabolite levels were corrected for frozen tissue weight to obtain total concentration, expressed as ng neurochemical/mg tissue weight.

### Data Analysis

Percent freezing data during fear learning (five trials) were analyzed with a repeated measures analysis of variance (R-ANOVA) test, with cognitive flexibility treatment/SPS condition as fixed effects. Fear extinction (30 trials) and extinction retention (10 trials), due to their length, were separated into an early phase (first half of trials) and late phase (second half of trials). Each phase was analyzed with a R-ANOVA test, with cognitive flexibility treatment and SPS condition as fixed effects. If an interaction effect was detected, a groupwise analysis was conducted comparing each group directly. Three rats were removed because they did not consistently show freezing behavior [two rats from the control group and one from the group exposed to cognitive flexibility and SPS, resulting in final group sizes of control (7), cognitive flexibility (7), cognitive flexibility and SPS (7), and SPS (9)]. Shock responses across the 5 shock-tone pairings during fear conditioning were also evaluated with a R-ANOVA. To evaluate the relationship between performance during the cognitive flexibility training and subsequent fear behavior during the extinction retention task, we used a R-ANOVA with cognitive flexibility performance as a fixed effect. Rats in the cognitive flexibility training were grouped with a median split for total percent correct during the cognitive flexibility training, to sort rats into high performers and low performers. For the group level neurochemical analysis, univariate general linear models were used with SPS and cognitive flexibility as fixed factors. If an interaction effect was detected, univariate general linear models were used to compare each group. If analytes were below threshold for detection for a tissue sample, that sample was excluded from analysis of that neurochemical for that region [ILC: 4 for NE, PLC: 7 for DA (a maximum of three per group, in the control group); striatum: one rat for DA, one rat HVA, three rats for 3MT]. In the ILC, nearly all rats were below threshold for DA, DOPAC, HVA, and 3MT, so these neurochemicals, and DOPAC:DA, were not analyzed for the ILC. For striatal NE, rats across all groups (21 total) were below the threshold for detection, so NE was not analyzed for the striatum. Analyses were run using IBM^®^ SPSS^®^ Statistics V. 24; values are reported as means ± standard error.

## Results

### Fear Learning

#### Fear Conditioning

**Figure 2:**
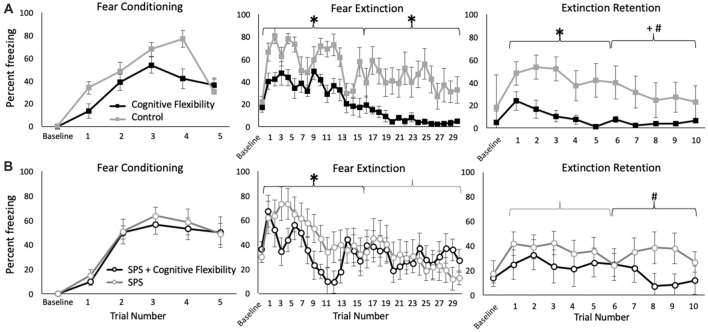
Effects of cognitive flexibility training on freezing during fear conditioning, fear extinction, and extinction retention testing in rats without **(A)** or with **(B)** prior exposure to single prolonged stress (SPS). Animals with cognitive flexibility training are indicated with black lines, animals with SPS are indicated with open circles. During fear conditioning, no group differences were detected. **(A)** In unstressed rats, cognitive flexibility training (black) enhanced extinction learning during the first and second half of the fear extinction learning trials (*p = 0.01, *p* = 0.04, respectively). Flexibility training also enhanced the retention of contextual information during extinction retention testing (*p < 0.05). **(B)** In rats exposed to traumatic stress, cognitive flexibility training increased extinction learning during the first half of extinction learning (*p = 0.05), and enhanced the rate of freezing attenuation during the second half of the extinction retention trials (treatment × time effect, ^#^*p* = 0.03).

Neither cognitive flexibility nor SPS affected freezing behavior during fear conditioning, and no interactions were detected (*p* > 0.05, Figure 2). Similarly, neither manipulation affected responsivity to the shock (*p* > 0.05, Supplementary Figure S1).

#### Fear Extinction Learning

Given prior evidence that SPS can have distinct effects on freezing behavior during the early and late phases of extinction testing, the first and second halves of the extinction testing were analyzed separately (Knox et al., 2012, 2016; Perrine et al., 2016). During the first half of extinction learning, cognitive flexibility enhanced extinction learning (main effect: *F*_(1,26)_ = 6.27, *p* = 0.02) whereas traumatic stress exposure decreased extinction learning over time (SPS × time effect: *F*_(1,26)_ = 2.19, *p* < 0.01). A groupwise analysis revealed that in unstressed rats, cognitive flexibility enhanced extinction learning (*F*_(1,15)_ = 11.82, *p* = 0.01, Figure 2A) and in trauma-exposed rats, prior cognitive flexibility training increased extinction learning in the early phase of the extinction trials compared with rats exposed to trauma alone (*F*_(1,13)_ = 4.34, *p* = 0.05, Figure 2B). Additionally, traumatic stress (SPS) exposure decreased the rate of extinction learning (SPS vs. control rats, both without cognitive flexibility training, SPS × time effect: *F*_(1,15)_ = 1.94, *p* = 0.03).

In the second half of extinction learning, cognitive flexibility enhanced extinction learning (main effect: *F*_(1,26)_ = 3.94, *p* = 0.05). There was a trend-level interaction between traumatic stress and cognitive flexibility training (*F*_(1,26)_ = 2.98, *p* = 0.09). A groupwise analysis revealed that in unstressed rats, cognitive flexibility enhanced extinction learning during the second half of the extinction testing (*F*_(1,15)_ = 6.11, *p* = 0.04, Figure 2A). In rats exposed to traumatic stress, cognitive flexibility training did not affect extinction learning in the late phase (*F*_(1,13)_ = 0.10, *p* = 0.76, Figure 2B). SPS exposure alone did not affect the second half of extinction learning trials (SPS vs. control rats, both without cognitive flexibility training, *F*_(1,15)_ = 0.08, *p* = 0.78).

#### Extinction Retention Testing

In the first half of the extinction retention testing, cognitive flexibility training enhanced the retention of contextual cues from fear extinction training (main effect: *F*_(1,26)_ = 7.26, *p* = 0.01). No other effects were detected in the first half of extinction retention testing. In the second half of extinction retention testing, cognitive flexibility training also enhanced retention (main effect: *F*_(1,26)_ = 5.88, *p* = 0.02) and this effect interacted with SPS exposure (SPS × cognitive flexibility × time effect: *F*_(1,26)_ = 5.47, *p* = 0.03). A groupwise analysis revealed that cognitive flexibility enhanced extinction retention under control conditions, in the absence of prior traumatic stress (*F*_(1,15)_ = 9.72, *p* = 0.01, Figure 2A). For rats that were exposed to traumatic stress, prior cognitive flexibility training enhanced extinction recall over time (cognitive flex × time: *F*_(1,13)_ = 2.97, *p* = 0.03, Figure 2B). Cognitive flexibility exposed rats with and without subsequent trauma exposure did not differ in either phase of the extinction retention testing (respectively, *F*_(1,13)_ = 2.63, *p* = 0.16, *F*_(1,13)_ = 3.53, *p* = 0.09). Traumatic stress (SPS) exposure attenuated the rate of change in freezing behavior in the second half of the extinction retention testing, which is congruent with prior findings that SPS impairs extinction retention (SPS vs. control rats, both without cognitive flexibility training, *F*_(1,15)_ = 2.87, *p* = 0.03).

**Figure 3:**
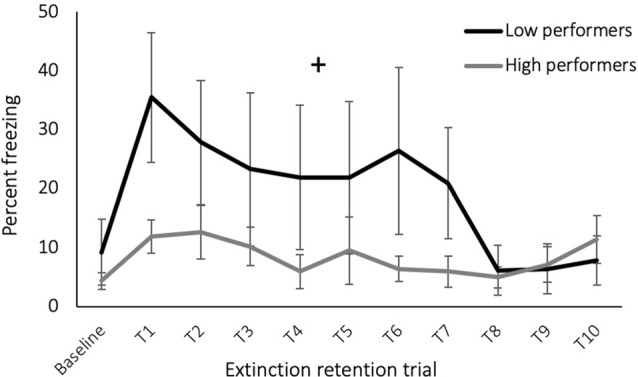
Freezing during extinction retention testing, groups separated by performance during reversal learning task of cognitive flexibility training. Rats with low reversal performance exhibited an increase in freezing across the trials (+ time × performance effect: *p* = 0.05).

When the results were examined for individual performance during the cognitive flexibility phases, rats with high performance during the reversal learning task demonstrated a greater rate of extinction retention during the extinction retention testing [Figure 3, reversal performance (percent of trials correct) × time: *F*_(1,13)_ = 1.93, *p* = 0.05] but did not have a main effect on freezing during extinction retention (effect of reversal performance: *F*_(1,13)_ = 0.02, *p* = 0.89). Performance during the other cognitive flexibility testing phases did not predict extinction retention (*p* > 0.05). This analysis included all rats exposed to the cognitive flexibility training, with and without subsequent exposure to SPS, based on our previous conclusion that cognitive flexibility affected fear learning behavior with or without trauma exposure and because of the variance required to detect individual level performance effects. Cognitive flexibility performance data are provided in Supplementary Figure S2.

### High-Pressure Liquid Chromatography (HPLC) Results

We analyzed levels of monoamines (DA and NE), and DA metabolites (DOPAC, 3MT, HVA), to understand monoamine signaling in brain regions regulating cognitive flexibility, fear learning processes, and aberrant fear responses in PTSD (Rauch et al., 2006; Milad et al., 2007, 2009; Klanker et al., 2013). Additional details for the results highlighted below, as well as for the infralimbic cortex which exhibited no treatment effects, are provided in Supplementary Table S1.

#### Prelimbic Cortex Monoamines and Metabolites

**Figure 4:**
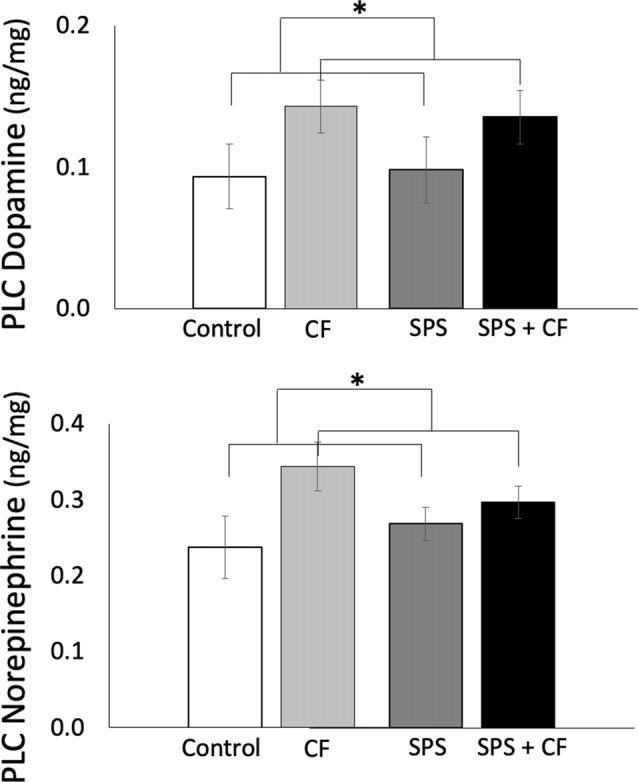
Exposure to cognitive flexibility treatment enhanced dopamine (DA; top panel) and NE (bottom panel) levels in the prelimbic cortex (PLC), detected as a main effect across animals that were and were not exposed to SPS, *p < 0.05.

Cognitive flexibility training elevated levels of DA and NE in the PLC (Figure 4; main effect across all groups, with and without exposure to SPS; DA: *F*_(1,26)_ = 5.49, *p* = 0.03, NE: *F*_(1,26)_ = 6.87, *p* = 0.01, no interactions were detected). As levels of DA metabolites are inherently low in the PLC, there were too many samples that did not reach minimum threshold to allow for reliable measurement of DA metabolites, such that tissue concentrations could not be estimated for the PLC.

#### Striatum Monoamines and Metabolites

In the striatum, there was an interaction between cognitive flexibility training and traumatic stress (SPS) for the DA metabolite 3MT (*F*_(1,26)_ = 5.09, *p* = 0.03), and levels of the 3MT metabolite were lower in animals exposed to both SPS and cognitive flexibility training compared with animals exposed to SPS alone (*F*_(1,15)_ = 6.83, *p* = 0.02), but cognitive flexibility treatment did not have a detectable effect on 3MT in the absence of SPS (*F*_(1,13)_ = 0.19, *p* = 0.67). Similarly, at the level of a trend there was an interaction between cognitive flexibility training and SPS exposure for the DA metabolite HVA (*F*_(1,26)_ = 2.90, *p* = 0.10), and HVA metabolite levels were lower in animals exposed to both SPS and cognitive flexibility training compared with animals exposed to SPS alone (*F*_(1,15)_ = 6.94, *p* = 0.02), but cognitive flexibility treatment did not have a detectable effect on HVA in the absence of SPS (*F*_(1,13)_ = 0.02, *p* = 0.90).

At the level of a trend, cognitive flexibility treatment lowered DOPAC levels in a comparison across all four groups (*F*_(1,26)_ = 3.58, *p* < 0.07), and, as with prior DA metabolites, DOPAC levels were lower in animals exposed to both SPS and cognitive flexibility training compared with animals exposed to SPS alone (*F*_(1,15)_ = 7.06, *p* = 0.02), but cognitive flexibility treatment alone did not have a detectable effect on DOPAC (*F*_(1,13)_ = 0.12, *p* = 0.73). Together, decreases in the DA metabolites DOPAC, HVA, and 3MT suggest that cognitive flexibility treatment buffered the effects of SPS on dopaminergic function in the striatum.

**Figure 5:**
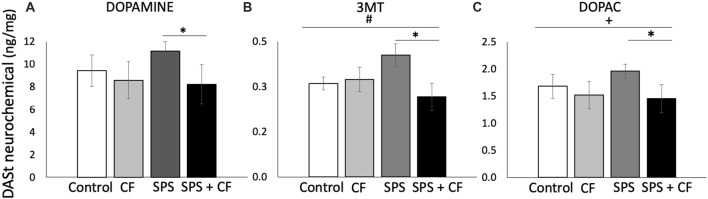
DA and its metabolites in the dorsal anterior striatum are affected by cognitive flexibility treatment. **(A)** DA levels were lower in animals exposed to both SPS and cognitive flexibility training compared with animals exposed to SPS alone (* indicates *p* = 0.04). **(B)** For the DA metabolite 3MT, there was an interaction between cognitive flexibility training and SPS exposure (^#^*p* = 0.03), and levels of the 3MT metabolite were lower in animals exposed to both SPS and cognitive flexibility training compared with animals exposed to SPS alone (*p = 0.02). Similarly, **(C)** at the level of a trend there was effect of cognitive flexibility training across all groups on the DA metabolite DOPAC (^+^*p* = 0.07), and DOPAC metabolite levels were lower in animals exposed to both SPS and cognitive flexibility training compared with animals exposed to SPS alone (*p = 0.02).

Because of* a priori* hypotheses and effects detected in DA metabolites, we compared DA levels in rats exposed to SPS alone or the cognitive flexibility followed by SPS. We found that DA levels were lower in animals exposed to both SPS and cognitive flexibility training compared with animals exposed to SPS alone (Figure 5; *F*_(1,15)_ = 5.04, *p* = 0.04). The ratio of a metabolite to its neurotransmitter can be determined as an indicator of turnover, thus tissue concentrations of DOPAC to DA were assessed as an estimate of DA turnover (Karolewicz et al., 2001; Cox et al., 2011). We found no effects of cognitive flexibility or SPS exposure on DOPAC:DA (main effect: *F*_(1,26)_ = 0.08, *p* = 0.78, *F*_(1,26)_ = 0.34, *p* = 0.57, respectively, no interaction detected, data in Supplementary Table S2).

## Discussion

Cognitive flexibility represents a unique cognitive ability that is linked to resilience and the ability to deal with unpredictable change (Kehagia et al., 2010). Cognitive flexibility varies across species, and has been proposed to increase with social complexity (Bond et al., 2007), foraging demands (Day et al., 1999), and environmental complexity and unpredictability (Belanger and Willis, 1996; Wright et al., 2010). Cognitive flexibility is highly variable within species, and highly flexible individuals also appear to be resilient to challenging conditions (Genet and Siemer, 2011; Laughlin et al., 2011; Miyake and Friedman, 2012; Romero-Martínez et al., 2013). Yet, to our knowledge, this is the first study to manipulate cognitive flexibility in the context of resilience to trauma. We examined whether cognitive flexibility training could buffer effects of traumatic stress on extinction retention, a hallmark deficit of PTSD, and corticostriatal monoamine signaling that maintains cognitive flexibility (Milad et al., 2008, 2009). We found that cognitive flexibility training enhanced extinction learning and can provide extinction retention benefits that remain after stress exposure. These benefits may be due to enhanced context processing skills conveyed by cognitive flexibility training that facilitate the recall of cues distinguishing the fear context from the safe context, or through an enhanced ability to discount old information from the fear context and acquire updated information from the safety context. To understand how individual differences in cognitive flexibility performance predict extinction retention ability, we compared performance during each cognitive flexibility training phase with extinction retention fear responses. We found that reversal learning ability predicts higher extinction retention, which prior evidence suggests is compromised by trauma (Milad et al., 2008, 2009; Knox et al., 2012, 2016; Perrine et al., 2016). Thus, reversal learning ability may facilitate prediction of individuals vulnerable to extinction retention deficits.

The precise mechanisms of cognitive flexibility in the context of trauma reactivity are underexplored. Prior research has demonstrated that both systemic increases in extracellular DA, *via* blockade of DA transporters, and moderate pharmacological increases in NE can have beneficial effects on cognitive flexibility, the current results expand on these prior findings to indicate that cognitive flexibility training can promote endogenous DA and NE increases within the PLC that may enhance context processing after trauma (Marek and Aghajanian, 1999; Volkow et al., 2002; Clatworthy et al., 2009; reviewed in Levrier et al., 2016). Moderate NE increases in the mPFC can facilitate cognitive flexibility by binding to α_2_-adrenergic receptors, however, excess NE binds to α_1_-adrenergic receptors, which can impair numerous executive functions (Arnsten et al., 1999; Carr et al., 2007). Both cognitive flexibility training and pharmacological blockade of α_1_-adrenergic receptors may modulate NE and DA-induced effects on postsynaptic excitatory currents to enhance decision making processes and fear extinction learning (Marek and Aghajanian, 1999; Knauber and Müller, 2000; Bernardi and Lattal, 2010; Schwager et al., 2014).

We found that cognitive flexibility training interacted with traumatic stress exposure to result in decreased striatal DA and DA metabolites, suggesting that cognitive flexibility training shapes corticostriatal monoamine signaling to modify trauma processing and cognitive processes during fear extinction learning and retention. These findings, in conjunction with an absence of change in the DOPAC to DA ratio, suggest that the entire DA system is upregulated with the overall DA metabolic rate remaining unchanged. The PLC and striatal DA results demonstrate region-specific dopaminergic changes resulting from combined cognitive flexibility training and traumatic stress; the region-specific DA patterns in the striatum are distinct from the behavioral results and PLC DA, which both emphasized the robustness of the effects of cognitive flexibility training with or without traumatic stress exposure. Thus, these results highlight the distinct roles of the PLC and striatum in regulating extinction learning and retention following traumatic stress. Given that cognitive flexibility performance is facilitated by systemic blockade of β-adrenergic receptors and striatal DA receptor availability (D2), but can be impaired by infusion of a DA receptor agonist into the striatum (Volkow et al., 1998; Beversdorf et al., 1999; Goto and Grace, 2005; Alexander et al., 2007), downstream effects of cognitive flexibility training on monoamine signaling may include changes in DA and NE receptor distribution. Overall, changes in striatal DA signaling can reciprocally modulate prefrontal monoamine signaling (reviewed in Klanker et al., 2013). Over-expression of striatal DA receptors can decrease PFC DA turnover and cause learning and memory deficits (Kellendonk et al., 2006; Bach et al., 2008). Further, suppression of tonic striatal DA release enhances signaling from the PFC to the nucleus accumbens in the striatum, whereas enhanced DA release shifts striatal signaling to hippocampal inputs (Goto and Grace, 2005). Overall, our results suggest that cognitive flexibility training can increase prefrontal DA, without increasing DA in the striatum, potentially to prioritize flexible decision making over “reflexive” decision making. Conversely, traumatic stress exposure does not affect prefrontal DA, but appears to increase striatal DA, potentially resulting in “reflexive” behavior. When cognitive flexibility training occurs prior to traumatic stress exposure, cognitive flexibility training attenuates effects of traumatic stress on DA in the striatum while retaining prefrontal DA and NE changes precipitated by cognitive flexibility training in isolation. Thus, the current results emphasize the importance of monoamine signaling in PLC and striatum in maintaining cognitive flexibility, and suggest that further elucidation of downstream effects of cognitive flexibility training on corticostriatal signaling could provide valuable insights into mechanisms maintaining cognitive flexibility.

Cognitive flexibility is deficient in numerous pathologies, including anorexia, bipolar disorder, and obsessive compulsive disorder (Chamberlain et al., 2006; Dickstein et al., 2007; Tchanturia et al., 2012, 2013). In a study isolating the effects of cognitive flexibility training on anorexia, Brockmeyer et al. (2014) found that cognitive flexibility training improved cognitive flexibility performance (set-shifting), which improved perceived coping with stress. The cognitive flexibility training model in Brockmeyer et al. (2014) utilized set-shifting training; the current results indicate that a cognitive flexibility training adapted to include reversal learning could further enhance coping and could have applications for the treatment of stress-linked psychopathologies. For the treatment of PTSD, current interventions leverage computer based cognitive skill trainings to supplement more conventional treatments (Rizzo et al., 2012; Bomyea et al., 2015; Khanna et al., 2015). Further, trauma-exposed individuals that show improvement in cognitive flexibility following a month of cognitive training exhibit clinical improvement and attenuated PTSD symptoms 6 months post-trauma, compared with trauma-exposed individuals that completed control trainings (Ben-Zion et al., 2018). The cognitive training model used in Ben-Zion et al. (2018) included one aspect of cognitive flexibility, set-shifting, as well as other complex cognitive skill trainings. Our data indicate that a cognitive flexibility training model that incorporates reversal learning could enhance existing post-trauma interventions and PTSD treatments, and may be beneficial for vulnerable populations with a high risk of encountering trauma.

A limitation of the current design is that assessment of monoamine levels occurred *ex vivo*, after all groups had completed fear learning assessments. Enman et al. (2015) found that SPS exposure can decrease levels of striatal DA and DA metabolites, DOPAC and HVA. Here, we did not find an effect of SPS on striatal DA, in the absence of an interactive effect with cognitive flexibility training. However, it has been shown that electric shock and fear learning procedures can enhance levels of striatal DA and DA metabolites in rats (Abercrombie et al., 1989; reviewed in Pezze and Feldon, 2004). Thus, a decrease in striatal DA induced by SPS could have been masked by subsequent fear learning procedures, emphasizing the role of striatal DA in aversive learning processes (Fadok et al., 2009). Overall, investigation of temporal monoamine changes following cognitive flexibility treatment, as well as investigation of downstream effects of changes in monoamine levels, could advance understanding of mechanisms that maintain cognitive flexibility. Further, additional control groups accounting for effects of novelty and cognitive stimulation, or the assessment of specific phases of the cognitive flexibility training, could help isolate the effects of cognitive flexibility. Additionally, although females were not studied here, evidence of sex differences in trauma reactions is robust (reviewed in Shansky, 2015; Bangasser and Wicks, 2017). Future studies elucidating the mechanism by which cognitive flexibility buffers effects of trauma should investigate sex-specific effects as well as effects on suites of psychopathological symptoms that have been characterized across the sexes. Although a main effect of SPS was not detected in the current experiment, effects of SPS on extinction retention freezing behavior were detected over time, and extensive prior evidence demonstrates that SPS (and PTSD) impair extinction retention (Milad et al., 2008, 2009; Knox et al., 2012, 2016; Chen et al., 2018). Thus, the authors feel the results demonstrate that cognitive flexibility training has the potential to be a meaningful non-invasive strategy to enhance wellbeing in the context of trauma and vulnerable populations.

Overall, our results demonstrate that cognitive flexibility training enhances extinction retention, a hallmark of PTSD, and PLC DA with or without subsequent traumatic stress exposure. Further, the current findings advance understanding of the role of monoamine signaling in cognitive flexibility by demonstrating that cognitive flexibility training can increase prelimbic DA and NE, and these effects are sustained after trauma exposure. Elucidating the mechanism by which cognitive flexibility modulates corticostriatal monoamine signaling could provide additional insights for the design of pharmacological interventions to mitigate adverse effects of trauma exposure. Given that individuals with high cognitive flexibility show reduced PTSD symptom severity and greater posttraumatic growth (Keith et al., 2015), cognitive flexibility training may have potential as an intervention for vulnerable populations or to supplement existing PTSD treatments.

## Data Availability

The raw data supporting the conclusions in the present manuscript will be provided to any researcher upon request.

## Author Contributions

LC and IL contributed to the design of the study. LC, KK and ML conducted the study. LC analyzed the data. LC, IL and SP wrote the manuscript.

## Conflict of Interest Statement

The authors declare that the research was conducted in the absence of any commercial or financial relationships that could be construed as a potential conflict of interest.

## Abbreviations

PTSD: posttraumatic stress disorder
SPS: single prolonged stress
DA: dopamine
NE: norepinephrine
DOPAC: 3,4-Dihydroxyphenylacetic acid
HVA: homovanillic acid
3MT: 3-Methoxytyramine.
