# Supplementary material for: Cognitive Flexibility Training Improves Extinction Retention Memory and Enhances Cortical Dopamine With and Without Traumatic Stress Exposure
Source: Front Behav Neurosci. 2019 Mar 1;13:24. doi: 10.3389/fnbeh.2019.00024 (PMC6406056; doi:10.3389/fnbeh.2019.00024)
Supplement: Supplementary file 1 [file Data_Sheet_1.docx]

**Supplementary figures:**


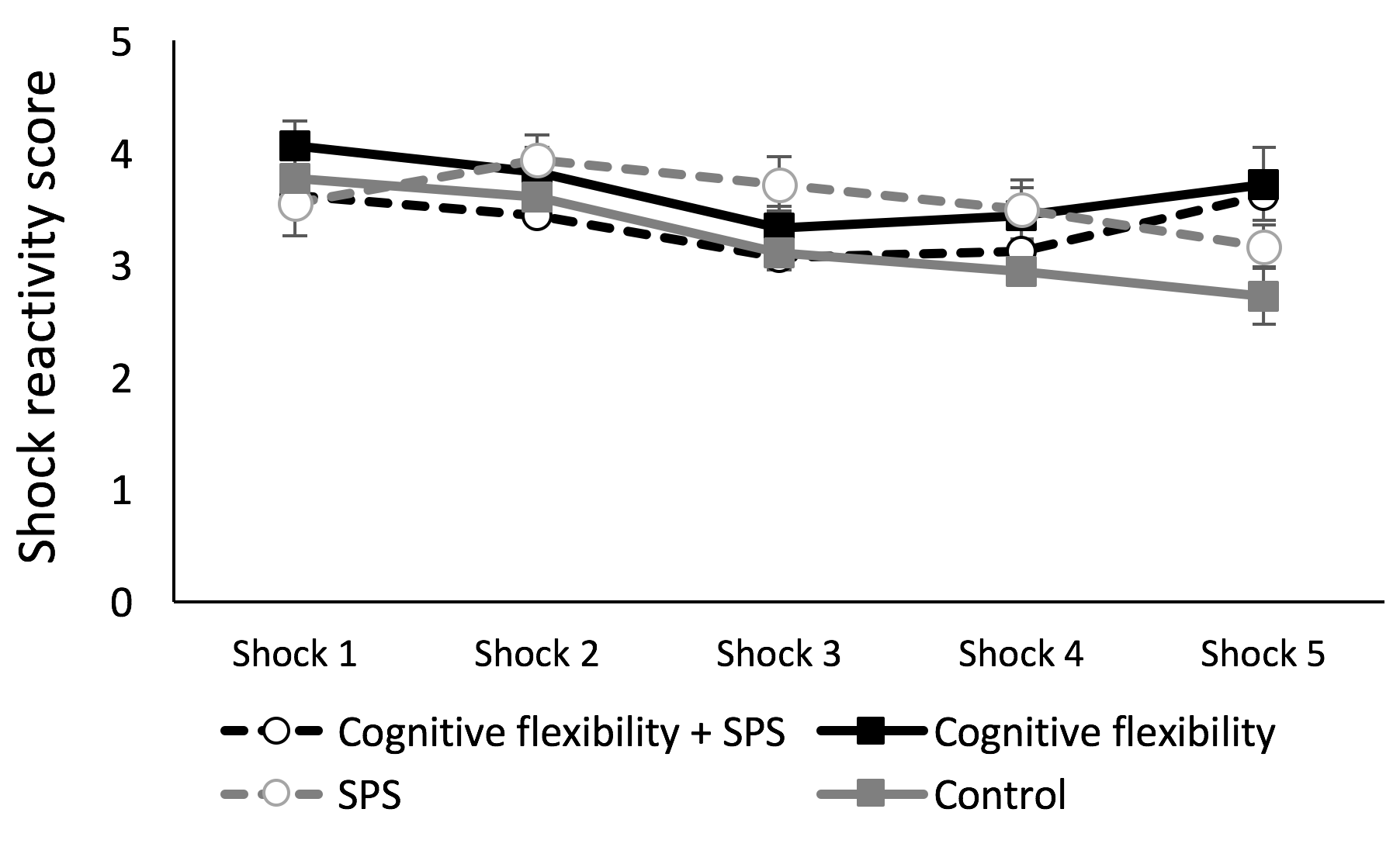


**Supplementary figure 1:** Neither the cognitive flexibility treatment nor SPS affected responsivity to the shock (F = 1.91, p = 0.15).

**
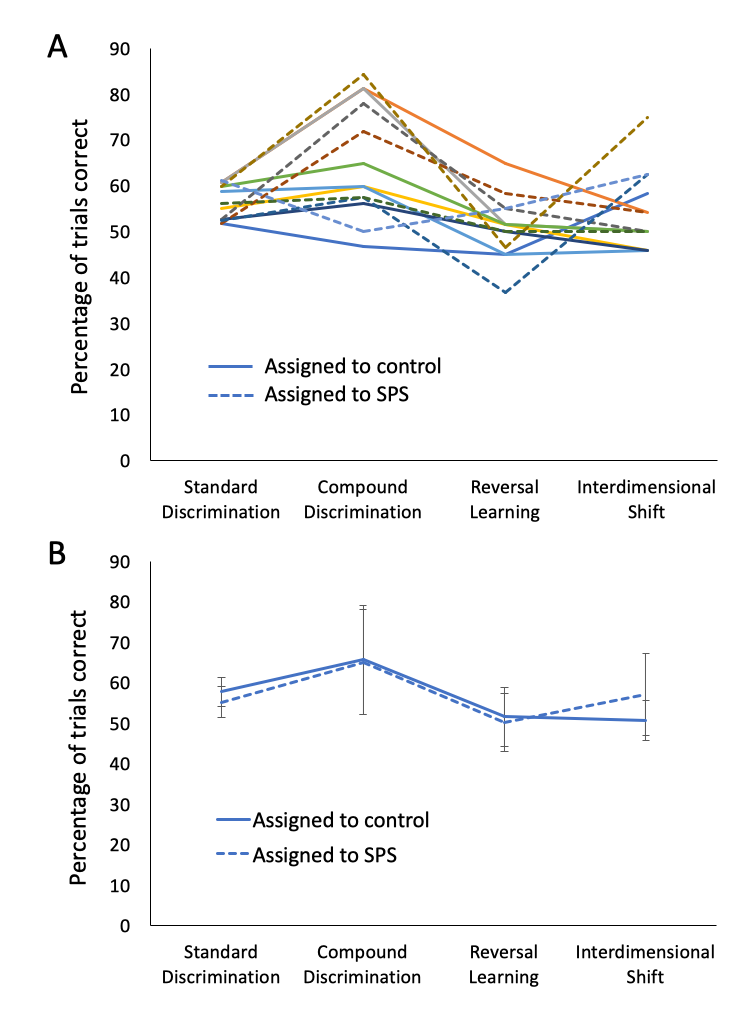
**

**Supplementary figure 2:** Total performance (percentage of trials correct) in each phase of cognitive flexibility training shown as (A) individual rats with uniquely colored lines and (B) group means. Rats that were then assigned to the single prolonged stress (SPS) group are shown with dashed lines, rats assigned to the control group are shown with solid lines. Within each phase, cognitive flexibility training continued until 80% of rats passed a learning criterion of six consecutive correct trials, to provide variance for determination of whether high or low performance affected potential benefits of the cognitive flexibility training. Rats were then separated into high and low performers, using total performance across all phases (median split), and half of the high and low performers were then subjected to either traumatic stress (single prolonged stress) or were maintained in control conditions.

**Supplementary table 1:** Monoamine and monoamine metabolite concentrations (ng/mg)

| Norepinephrine concentration (ng/mg) | | | | |
| --- | --- | --- | --- | --- |
| Brain region: | Cognitive Flexibility | Cognitive Flexibility + SPS | Control | Control + SPS |
| Infralimbic cortex | 0.42+0.04 | 0.34+0.05 | 0.35+0.03 | 0.34+0.04 |
| Prelimbic cortex | 0.34+0.03 | 0.29+0.02 | 0.24+0.04 | 0.26+0.02 |

| Dopamine concentration (ng/mg) | | | | |
| --- | --- | --- | --- | --- |
| Brain region: | Cognitive Flexibility | Cognitive Flexibility + SPS | Control | Control + SPS |
| Prelimbic cortex | 0.14+0.01 | 0.13+0.01 | 0.09+0.005 | 0.10+0.005 |
| Striatum | 8.58+1.66 | 8.23+1.74 | 9.45+1.39 | 11.18+0.82 |

| 3,4-Dihydroxyphenylacetic acid **(**DOPAC)  concentration (ng/mg) | | | | |
| --- | --- | --- | --- | --- |
| Brain region: | Cognitive Flexibility | Cognitive Flexibility + SPS | Control | Control + SPS |
| Striatum | 1.52+0.25 | 1.45+0.26 | 1.68+0.22 | 1.96+0.13 |

| Homovanillic acid (HVA) concentration (ng/mg) | | | | |
| --- | --- | --- | --- | --- |
|  | Cognitive Flexibility | Cognitive Flexibility + SPS | Control | Control + SPS |
| Striatum | 1.14+0.13 | 1.01+0.16 | 1.15+0.16 | 1.27+0.08 |

| 3-Methoxytyramine (3MT) concentration (ng/mg) | | | | |
| --- | --- | --- | --- | --- |
|  | Cognitive Flexibility | Cognitive Flexibility + SPS | Control | Control + SPS |
| Striatum | 0.32+0.04 | 0.27+0.04 | 0.31+0.02 | 0.41+0.04 |

**Supplementary table 2:** 3,4-Dihydroxyphenylacetic acid **(**DOPAC) to dopamine (DA) tissue concentration ratio (ng/mg)

| DOPAC:DA | | | | |
| --- | --- | --- | --- | --- |
|  | Cognitive Flexibility | Cognitive Flexibility + SPS | Control | Control + SPS |
| Striatum | 0.20+0.02 | 0.20+0.02 | 0.19+0.02 | 0.18+0.01 |

**Supplementary methods:**

*Fear Conditioning, Fear Extinction, Extinction Retention*

Trials were conducted in sound-attenuating experimental chambers (30x24x21cm, MED Associates, VT), connected to an interface controlling experimental contingencies using MedPC software. The chamber floor was made of stainless steel rods (4mm diameter), connected to a shock source that delivered a 1 second, 1mA shock, which occurred during fear conditioning immediately after an acoustic tone. Tones were emitted from a speaker inside the chamber (10 second 1kHz 80dB). Each chamber was also equipped with a 15W light and a fan that provided a 65dB white noise to distinguish the fear conditioning context from a second context for fear extinction and extinction retention. The two contexts had distinct visual, olfactory, and tactile cues.

*Fear conditioning context cues*: Rats were transported to experimental chambers in black plastic transfer containers and the testing room was lit by red light; ammonium hydroxide vapors (1%) filled the experimental chamber and testing room. Additionally, doors of the sound-attenuating boxes were left open, and the chamber lights and fans were left off.

*Fear extinction and extinction retention context cues*: Rats were transported to experimental chambers in white plastic transfer containers with clean bedding and the testing room was lit by standard white light; acetic acid vapors (1%) filled the experimental chamber and testing room. Additionally, doors of the sound-attenuating boxes were closed, and the chamber lights and fans were on.

Baseline movement was recorded for 3 minutes before cues were presented. For fear conditioning, rats were presented with five tones as the conditioned stimulus that co-terminated with a foot shock as unconditioned stimulus. For fear extinction and extinction recall, rats were presented with 30 or 10 conditioned stimulus tones, respectively, without paired shocks. Tones lasted 10 seconds and time between each conditioned stimulus was 1 minute. For all three tests, animals were transported to the testing room and left to acclimate for 10 minutes in their transport boxes before testing. Transport boxes and testing chambers were cleaned and dried between animals.
